# Biometrical and histomorphometrical changes of testis in the dynamics of postnatal ontogenesis from birth to puberty of Black Bengal goat

**DOI:** 10.5455/javar.2023.j674

**Published:** 2023-06-30

**Authors:** Md. Royhan Gofur, Md. Sheikh Sadi, Shabnaz Aktar, Afia Khatun, Md. Abdul Awal, Md. Emtiaj Alam, Shah Md. Abdur Rauf, Koiochi Matsuo

**Affiliations:** 1 Department of Veterinary and Animal Sciences, University of Rajshahi, Rajshahi, Bangladesh; 2 Department of Anatomy and Histology, Bangladesh Agricultural University, Mymensingh, Bangladesh; 3 Laboratory of Cell and Tissue Biology, Keio University School of Medicine, Tokyo, Japan

**Keywords:** Biometry, postnatal ontogenesis, spermatogenesis, puberty, black Bengal goats

## Abstract

**Objectives:**

The study aimed to account for baseline biometrical and histomorphometric testicular changes in Black Bengal goats during postnatal development.

**Materials and Methods:**

Black Bengal goats, divided into group I of VII; day 0; 1, 2 weeks; 1, 2, 4, and 6 months of age, respectively, were used in this study.

**Results:**

The biometrical and histomorphometric values of the testis varied significantly (*p* < 0.05) from postnatal 1–2 months. From day 0 to 2 months, seminiferous tubules, called sex cords, contained simply peripherally placed Sertoli cells and centrally placed gonocytes. Gonocytes, positioned in the center, moved centrifugally in the direction of the basement membrane of sex cords with the advancement of age, transformed into prespermatogonia, and were distributed among the Sertoli cells at the edge of sex cords that make up the basal cell layer in nearly all of the seminiferous tubules by 2 months after birth. Initiation of spermatogenesis, i.e.*, *stratification and lumination of seminiferous epithelium, took place in the 4th months. At 6 months, all types of spermatogenic cells had been identified. The onset of puberty, i.e., the establishment of spermatogenesis, was noticed to have been established at 6 months of postnatal age in Black Bengal goats, as shown by the spermatozoa that were adhered to the ad luminal border of the Sertoli cells and also in the tubular lumen.

**Conclusion:**

This research is the first to document the varying biometrical and histomorphometric measurements of the testis in Black Bengal goats from birth to puberty.

## Introduction

Among the goat genetic resources available worldwide, the Black Bengal goat holds a significant position. In Bangladesh, the Black Bengal goat ranks second in terms of economic importance among domestic animals and is employed as a strategy to encourage sustainable livelihoods in rural Bangladesh [1]. One of the main issues limiting goat production in Bangladesh is the severe lack of genetically superior bucks across the entire nation. Superior buck selection appears to be a crucial and alternative strategy for boosting the production potential. Understanding the fundamental morphometric parameters of the reproductive organs is very helpful when evaluating the breeding soundness and probable fertility of breeding males. In particular, testicular biometrics during postnatal development are important aspects in determining sexual maturity (puberty), superior buck selection, and interpretations about spermatogenesis [2].

In the course of the postnatal development of the testis, somatic and germ cells proliferate and differentiate in order to produce the first cycle of spermatogenesis and establish a basis for future continuous sperm production [3]. In the early postnatal period, the gonocytes moved centrifugally from the center to the basement membrane, where they were transformed into spermatogonial stem cells and subsequently produced differentiated spermatogonia [4]. The spermatogenesis is fully established, or the start of puberty, after the seminiferous tubules have fully grown, as evidenced by the appearance of spermatozoa for the first time in the lumen of the seminiferous tubules [5]. When sex cord/seminiferous tubule development is finished in Black Bengal goats, it is unclear; however, in contrast to other breeds, this breed has a large genetic heritage and has evolved under extremely diverse environmental conditions, so certain variances are to be expected. Moreover, precise knowledge of the morphometrical traits of the testis from birth until the start of puberty is needed for the efficient reproductive management of economically important animals.

To understand the postnatal growth and maturation of the genital system or organs, postnatal biometrical and histomorphometrical research until puberty is essential. Sporadic reports on the biometry and histomorphometry of the testis of Assam goats [6], Balami goats, Uda goats, Yankasa goats [7], Gaddi goats [8], and Japanese Tokara goats [9] are available. Despite being the sole breed of goat in Bangladesh, the Black Bengal, no systematic research on the testicular postnatal development of the Black Bengal goat breed has been done. This study documents the biometric and histomorphometric changes in the testis of Black Bengal goats from birth to puberty sequentially. This research is the first to document the postnatal growth of the testes in the Black Bengal goat until puberty, which will be helpful to anatomists, cell biologists, and thieriogenologists.

## Materials and Methods

Black Bengal goats, bought from local markets near Rajshahi University (*n = *21), varying in postnatal age from birth (day 0; d0) to 6 months, were reared under standard farmhouse circumstances. The testes of these goats were used for biometrical and histomorphometrical analyses. There were seven age groups (*n = *3) for the goats, namely group-I (day 0; d0), group-II (1 week), group-III (2 weeks), group-IV (1 month), group-V (2 months), group-VI (4 months), and group-VII (6 months). Birth records were accurately kept, allowing us to determine the age of the goats under study. Animal experiments were conducted according to the guidelines set by the Institutional Animal, Medical Ethics, Biosafety, and Biosecurity Committee of the University of Rajshahi, Bangladesh (Memo No. 293(13)/320/IAMEBBC/IBSc). After induction of anesthesia and cutting the scrotum on the lateral side, the testes were collected. Left testes were subsequently fixed in a 10% formalin solution for histomorphometrical study, and right testes were used for the biometrical study.

According to the method used by Gofur et al. [10] for a histomorphometrical investigation, all of the formalin-fixed tissues were prepared into paraffin slices and stained with a standard hematoxylin and eosin stain. Stained sections were inspected using a compound microscope at magnifications of 10, 20, and 40 (Kruss, Germany) with a digital camera. Some stained testis sections were scanned by the NanoZoomer S60 digital whole slide scanner (C13210-01, Hamamatsu, Japan). The scanned NanoZoomer digital images were viewed by the NDP.view2 Viewing software (U12388-01). The data were statistically analyzed using statistical software available online (https://astatsa.com/OneWay_Anova_with_TukeyHSD/). The data were shown as mean ± SE. One-way analysis of variance, followed by Turkey honestly significant difference *post-hoc* analysis, was used to assess variations in biometrical and histomorphometrical values between the various postnatal ages.* p* values under 0.05 were regarded as significant.

## Results

### Biometry of postnatal developing testis of Black Bengal goat

**Figure 1.**
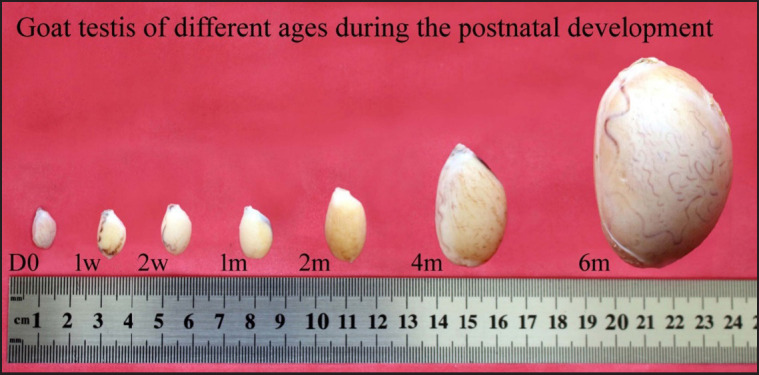
Gross images of Black Bengal goat testes at various postnatal ages, from birth to puberty. d0, at birth or day 0; 1 w, one week; 2 w, two weeks; 1 m, one month; 2 m, two months; 4 m, four months; 6 m, six months of postnatal age.

Testes from Black Bengal goats at various postnatal ages were measured for length, width, and weight in order to evaluate the relative biometry of testes during postnatal development. Representative postnatal testicular development figures of various age groups of Black Bengal goats and their biometric measurements (length, width, and weight) are shown in Figure 1 and Table 1, respectively. During the age progression of goat kids, the testicles grew in length, breadth, and weight. Different postnatal age groups had distinct testicular biometric parameters. Up to 2 weeks of age, biometrical values within age groups (d0, 1 and 2 weeks) that were insignificant suggested that the testicular growth was slow, and then until puberty, rapid testicular development was seen, which was demonstrated by a substantial difference (*p < *0.05) in biometrical values between age groups (1, 2, 4, and 6 months). Compared to birth, the testicular length and breadth expanded by 5 to 6 times, and the weight increased by 100 times at puberty.

**Table 1.** Testicular biometric measurements (mean ± SE) at various postnatal ages in Black Bengal goats.

Postnatal ages	Length (cm)	Breadth (cm)	Weight (gm)
At birth or day 0 (d0)	1.18 ± 0.053^a^	0.82 ± 0.062^a^	0.40 ± 0.047^a^
1 week (1 w)	1.37 ± 0.057^ab^	0.93 ± 0.058^ab^	0.63 ± 0.037^a^
2 weeks (2 w)	1.63 ± 0.042^b^	1.03 ± 0.047^ab^	0.94 ± 0.045^a^
1 month (1 m)	1.79 ± 0.079^b^	1.13 ± 0.054^b^	1.59 ± 0.140^b^
2 months (2 m)	2.48 ± 0.137c	1.45 ± 0.103^c^	3.32 ± 0.207^c^
4 months (4 m)	3.79 ± 0.163^d^	2.52 ± 0.132^d^	12.23 ± 0.789^d^
6 months (6 m)	6.57 ± 0.239^e^	4.12 ± 0.118^e^	41.02 ± 2.846^e^

Note: Significant variations (*p* < 0.05) between postnatal ages are indicated by values in a column with various superscripts.

### Histomorphometry of postnatal developing testis of Black Bengal goats

**Figure 2.**
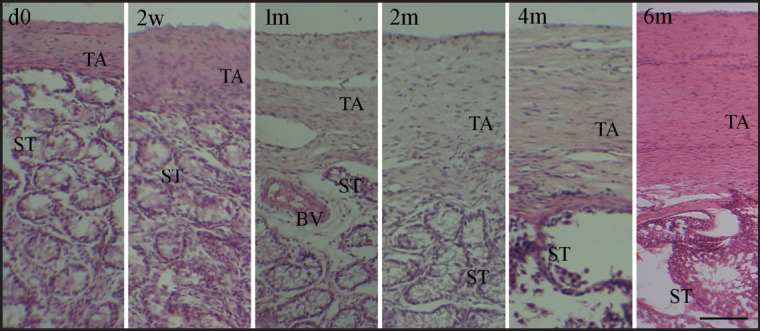
Histological micrographs depicting the tunica albuginea of the Black Bengal goat testicles of various postnatal ages, from birth to puberty. Hematoxylin and eosin stains were used to stain the sections. During the postnatal development, an increasing trend in the thickness of tunica albuginea with age was detected. From 1 month (1 m) of age and beyond, a substantial variation in thickness among postnatal age groups was noted. d0, day 0; 1 w, 1 week; 2 w, 2 weeks; 1 m, 1 month; 2 m, 2 months; 4 m, 4 months; 6 m, 6 months of postnatal age; TA, tunica albuginea; ST, seminiferous tubules; BV, blood vessel; scale bar 100 μm.

The testis of a Black Bengal goat underwent postnatal development, and histomorphometric analyses indicated changes in the seminiferous epithelium, seminiferous tubule diameter, and tunica albuginea thickness at various ages. With the advancing age of Black Bengal goats, a progressive increase in tunica albuginea thickness was seen during postnatal development (Fig. 2). Although the tunica albuginea thickness changed with age groups of goats, significant (*p < *0.05) differences across age groups were not seen until 1 month after birth (Table 2). Tunica albuginea saw a roughly 2.5-fold increase in thickness from birth (day 0) to the 6th month.

The parenchyma of the testis at birth or in day 0 goat kids consists of seminiferous tubules scattered throughout the interstitial tissue or stroma. The tubular diameter was 37.56 ± 3.62 μm (Table 2). Most of the tubules were spherical, with only a few of the convoluted variety. The tubules contain no lumen; hence, these tubules were called the sex cords. The interior of the sex cords was filled with acidophilic ground materials. These solid sex cords featured large central germ cells (gonocytes) with a spherical centrally positioned nucleus and acidophilic cytoplasm, as well as peripheral Sertoli cells (lying on the basement membrane) with a generally ovoid-shaped nucleus and light cytoplasm (Fig. 4A). Each sex cord had a distinct basement membrane encircled by several layers of elongated peritubular cells, known as myoid cells (Fig. 4A). In the stroma, the area between the sex cords, there were numerous interstitial or stromal cells, including Leydig cells (Fig. 4A).

With the growing age of the goat kids, the dimensions of the sex cords, or seminiferous tubules, expanded. Seminiferous tubules in a pubertal buck had a diameter of 190.89 ± 9.38 μm, which was around five times larger than those at birth. Although seminiferous tubule diameter varied among age groups, only after 2 months of age were significant variations (*p < *0.05) across age groups noticed (Table 2). Moreover, postnatal development was accompanied by a steady decrease in stromal cells as well as an increase in tubular convolution and decreasing intertubular (stromal) space with advancing age in Black Bengal goats.

**Table 2.** Testicular histomorphometric parameters (mean ± SE) at various postnatal ages in Black Bengal goats.

Postnatal ages	Thickness of tunica albuginea (μm)	Breadth of seminiferous tubules (μm)
At birth or day 0 (d0)	118.75 ± 7.26^a^	37.56 ± 3.62^a^
1 week (1 w)	144.13 ± 10.93^ab^	45.78 ± 4.32^a^
2 weeks (2 w)	161.63 ± 11.30^abc^	52.11 ± 5.26^ab^
1 month (1 m)	178.75 ± 9.25^bcd^	60.56 ± 4.94^ab^
2 months (2 m)	199.13 ± 10.79^cd^	74.33 ± 7.03^b^
4 months (4 m)	223.63 ± 8.78^d^	162.44 ± 10.09^c^
6 months (6 m)	288.13 ± 10.17^e^	190.89 ± 9.38^d^

Note: Significant variations (*p* < 0.05) between postnatal ages are indicated by values with different superscripts in a column.

**Figure 3.**
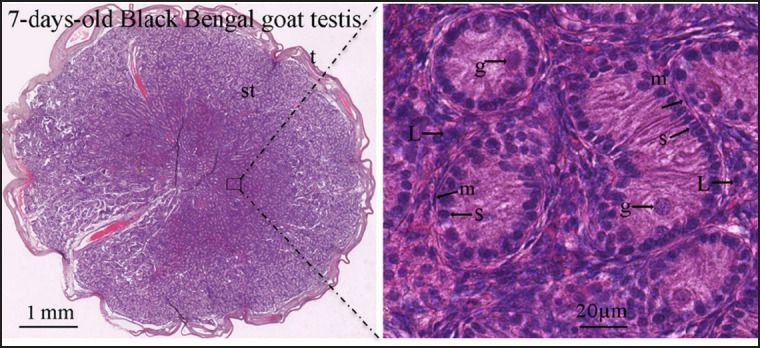
NanoZoomer digital images showing the histological architecture of 7-day-old Black Bengal goat testis. Testicular sections were stained with hematoxylin and eosin stains, and imaged using a NanoZoomer S60 digital whole slide scanner (C13210-01, Hamamatsu, Japan). NDP.view2 Viewing software (U12388-01)was used to view the scanned NanoZoomer Digital Images. t, tunica albuginea; st, seminiferous tubules; m, myoid cell; g, gonocyte; s, Sertoli cell; L, stromal cells with Leydig cells.

**Figure 4A–G.**
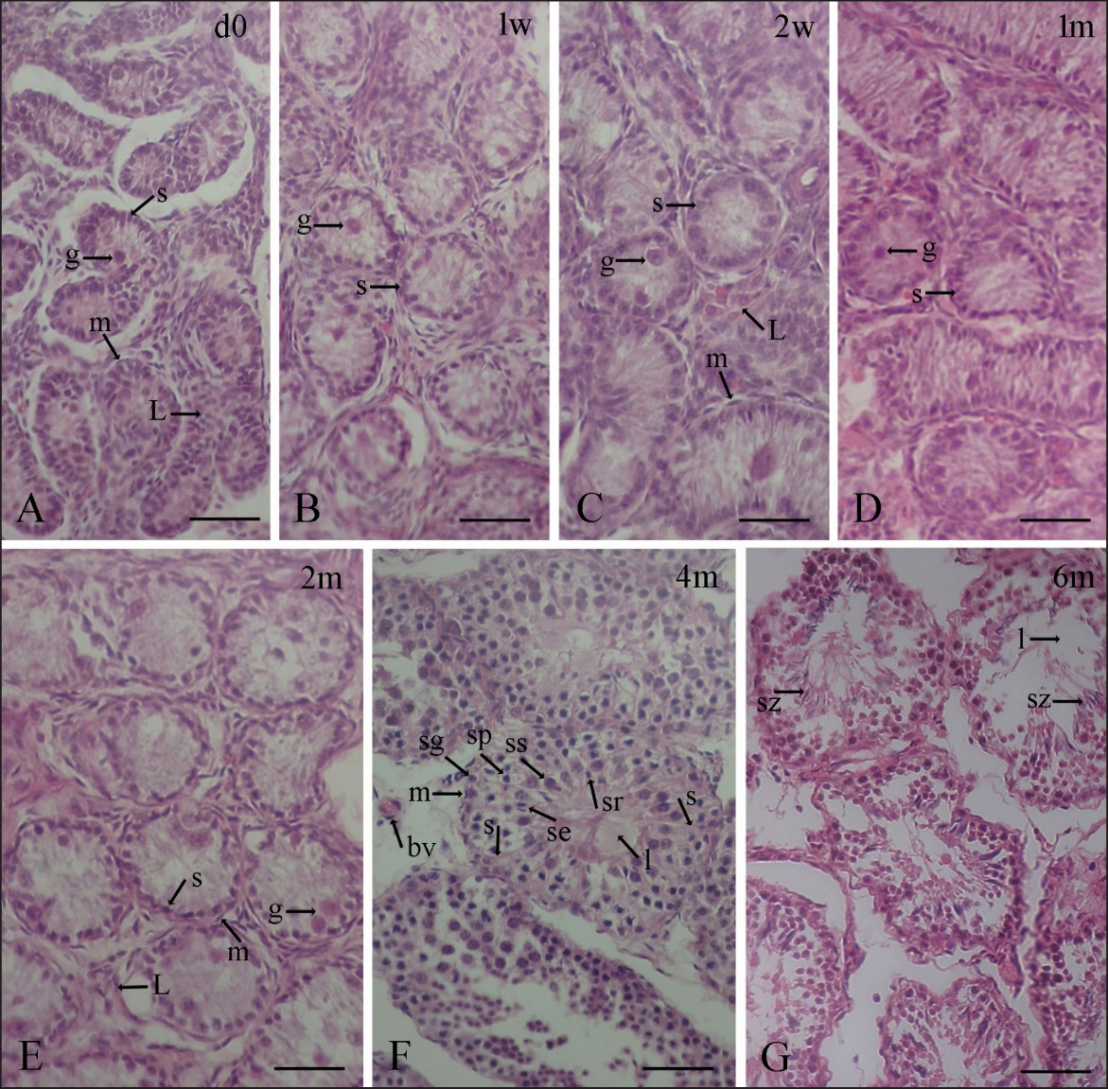
Histological images depicting the postnatal development of the seminiferous tubules in Black Bengal goats. Hematoxylin and eosin stains were used to stain the sections. Only Sertoli cells (located in the periphery) and gonocytes (located in the center)/prespermatogonia (located amid the Sertoli cells) with no lumen were present in the seminiferous tubules or sex cords at birth (day 0) to two months (2 m). The tubules of the four (4 m) and six (6 m) months olds have a distinct lumen. About 4 months after birth, seminiferous tubules with stratified seminiferous epithelium containing the spermatogonia, primary and secondary spermatocytes, round spermatids, and elongated spermatids adhered with Sertoli cells at their ad luminal border were seen. About 6 months after birth, seminiferous tubules contained all types of cells of the spermatogenic lineage including spermatozoa adhered with Sertoli cells at their ad luminal border and in the lumen suggesting the start of puberty in Black Bengal goats. d0, day 0; 1 w, 1 week; 2 w, 2 weeks; 1 m, 1 month; 2 m, 2 months; 4 m, 4 months; 6 m, 6 months of postnatal age; s, Sertoli cell; g, gonocyte; pg, spermatogonia; ps, primary spermatocytes; ss, secondary spermatocytes; sr, round spermatids; se, elongated spermatids; sz, spermatozoa; m, myoid cell; L, stromal cells with Leydig cells; bv, blood vessel; l, lumen; scale bar 50 μm.

**Figure 5.**
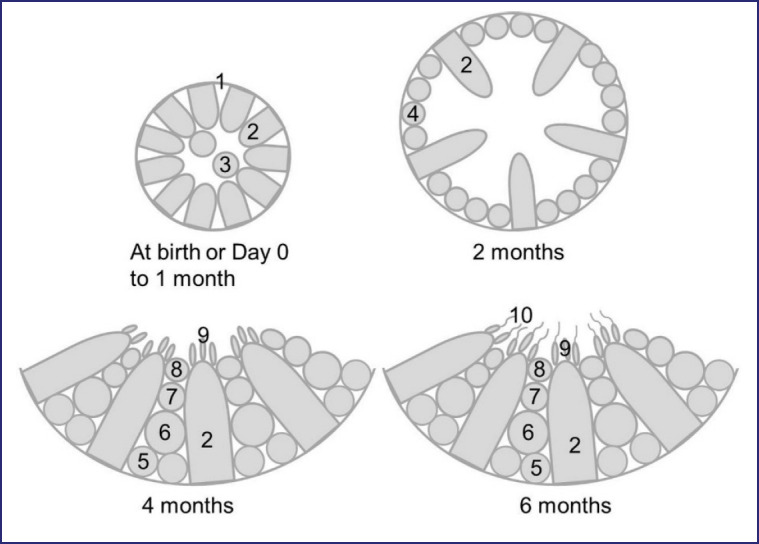
Schematic depictions demonstrating the overall postnatal developmental patterns of seminiferous tubules in the testis of Black Bengal goats. 1, basement membrane; 2, Sertoli cell; 3, gonocyte; 4, prespermatogonia; 5, spermatogonia; 6, primary spermatocyte; 7, secondary spermatocytes; 8, round spermatids; 9, elongated spermatids; 10, spermatozoa.

Until 1 month of age, seminiferous tubules had a structure resembling that of day 0, but as age progressed, gonocytes began to migrate toward the tubular basement membrane, which later became spermatogonia situated among the Sertoli cells at the periphery of tubules and conforming the basal cell layer of seminiferous tubules by 2 months postnatal age (Figs. 3 and 4B–E). After 4 months of postnatal age, the seminiferous tubules were shown to have a lumen in the middle (Fig. 4F). About 4 months of age, seminiferous tubules with stratified seminiferous epithelium containing the spermatogonia, primary, and secondary spermatocytes, round spermatids, and elongated spermatids adhered with Sertoli cells at their ad luminal border were seen (Fig. 4F). About 6 months after birth, seminiferous tubules contained all types of cells of the spermatogenic lineage, including spermatozoa, adhered to Sertoli cells at their ad luminal border and in the lumen (Fig. 4G). The lumen of a few seminiferous tubules from testes that were 4 and 6 months old had a small number of disconnected, degenerated, or apoptotic cells. The lumen of seminiferous tubules containing spermatozoa, which was revealed by histological studies, suggested that Black Bengal goats reached puberty 6 months after birth. Figure 5 demonstrates an overview of postnatal testicular (seminiferous tubule) development in Black Bengal goats.

## Discussion

To understand the development and expansion of the male genital system, it is vital to conduct postnatal developmental research on male genital organs at different ages, especially the primary organ (the testis) and its excretory duct system [11]. In order to monitor the testis for normality and estimate its capacity to produce sperm, gross and microscopic parameters of the testis are crucial variables. In particular, it has been claimed that testicular size or weight is a reliable predictor of sperm production in the present and the future in a variety of animal species [2]. With increasing age, the biometrical values of the Black Bengal goat testis in the current study greatly increased, which supports previous observations [12,13] in different documented goat breeds. We observed that the testis development of the Black Bengal goat was slower in the early postnatal phase and quicker between 4 and 6 months of age, around the time when spermatogenesis starts. The postnatal testicular development closely resembles that of the Yiling goat that Bo et al. [14] observed. The initial sluggish phase of testicular growth is characterized by low serum testosterone concentrations and high levels of androstenedione, whereas the rapid growth phase is characterized by high testosterone and low levels of androstenedione [15].

Different postnatal age groups had distinct testicular biometric parameters. Moreover, testicular weight did not alter significantly from birth (day 0) to 1 month of age (*p > *0.05), but from 2 months until puberty (6 months), it greatly increased (*p < *0.05). The biometrical results of the current investigation are congruent with those of earlier studies (in Yiling goats by Bo et al. [14]; in Gaddi goats by Pathak et al. [13]) in goats. The findings of the current investigation concur with those of Sadi and Gofur [16] in indigenous sheep and Gofur et al. [17] in indigenous bulls. We also found some inconsistencies with previous studies; testicular size and weight were less than those seen in West African Dwarf bucks by Olurode et al. [18], Gaddi goats by Pathak et al. [13], and higher than those in Black Bengal goats by Kabiraj et al. [19]. Breed variances, the physical condition of the selected goats, agroclimatic conditions, nutritional level, housing, and other management or experimental procedures could all contribute to the discrepancies.

Histologically, the testis is covered by the fibrous tissue covering of the testis called the tunica albuginea, and the parenchyma is mainly made up of seminiferous tubules. Gonocytes, or primordial germ cells, that move from the yolk sac into the major sex cords give rise to the testis. Eventually, the major sex cords transform into testicular cords (seminiferous cords), which are bordered by seminiferous epithelium [20]. During postnatal development until puberty, a pattern of steadily increasing thickness was seen in the tunica albuginea. A similar discovery was made with Assam goats [6]. However, Gofur et al. [10] noted a progressive reduction in tunica albuginea thickness in post-pubertal bulls, indicating the tunica albuginea increases in thickness up to puberty and/or animal maturity, at which point it begins to decline and maintains its thickness.

With the progression of age, the tubular diameter likewise demonstrated a similar upward trend as recorded in the Assam goat [6] and Tokara goat [9]. A rise in the area that seminiferous tubules occupy in the testis is linked to testicular growth. When compared to the parenchyma (35%) in newborn kid testes, the interstitial tissue/stroma (area/volume) was much higher (65%), whereas, in pubertal animals, it maintained this ratio at 13:87 [21]. This suggested that the parenchyma would expand more quickly in order to produce enough spermatozoa for breeding goats at puberty. The dramatic rise in seminiferous tubule diameter around puberty is responsible for this expanded parenchyma. After 2 months of age, the current investigation found a considerable increase in tubular diameter, and more specifically, between 2- and 4-month-old goats and between 4- and 6-month-old goats indicated rapid growth of the tubules at the onset of spermatogenesis and before the onset of puberty in Black Bengal goats, respectively. We noticed a progressive decline in interstitial cell number, contrasting with the gradual increase in seminiferous tubule occupancy in the parenchyma. A progressive reduction in intertubular space, along with a reduction in stromal cell number, was also recorded in Assam goats [6], Gaddi goats [21], and Institute of Cancer Research mice [20] when they were developing after birth.

The seminiferous tubules, or sex cord, had just two cell types (Sertoli cells and germ cells, or gonocytes) at birth (d0) and for the first 2 months after birth. At birth, enormous spherical gonocytes were positioned in the center, while Sertoli cells were positioned peripherally, resting on the basement membrane. As the goat kids became older, these gonocytes began to migrate toward the sex cord’s basement membrane, at which point they were known as prespermatogonia, which were positioned amid the Sertoli cells at the periphery of the sex cords, composing the basal cell layer in nearly all of the seminiferous tubules by the 2-month postnatal age of the Black Bengal goat. Sarma and Devi [22] observed the location of prespermatogonia among Sertoli cells, and the centrifugal movement of gonocytes followed a similar pattern in Assam goats. Similar gonocyte centrifugal movements that result in prespermatogonia have also been seen in other domestic animals [4]. Furthermore, Shima [23] and Nagano et al. [24] described that age-related migration of the core gonocytes to the sex cord’s periphery, followed by a succession of mitotic divisions leading to three proliferative stages, resulted in the formation of prespermatogonia, which produced spermatogonia in later generations. In seminiferous tubules of Black Bengal goats, lumination was first noticed at 4 months of age, far later than in Assam goats. Nonetheless, it has been noted that domestic animals exhibit a range in the age at which luminization can be established in the seminiferous tubules.

Four months after birth, seminiferous tubules showed signs of stratification. This stratified epithelium included spermatogonia, primary and secondary spermatocytes, and spherical and elongated spermatids, and the latter adhered to Sertoli cells at their ad luminal border. Four-month-old goats were found to have begun the process of spermatogenesis since spermatocytes were first detected in the seminiferous epithelium at this time. The beginning of spermatogenesis in Assam goats was also noted by Sarma and Devi [22] to occur about 4 months after birth. With the start of spermatogenesis, the testis undergoes a significant increase in size [25]. At 4 months postnatal age, we also discovered a sharp and considerable increase in the tubular diameter of Black Bengal goats*.*

The seminiferous tubules of a 6-month-old testis included all spermatogenic cell types, indicating that seminiferous tubule development was completed at this time. The current study verified that in Black Bengal goats, puberty began at 6 months postnatal age based on the initial appearance of spermatozoa attached to the ad luminal border of Sertoli cells and the lumen of the seminiferous tubules*. *A histological investigation of mice at 5 weeks old revealed the earliest evidence of spermatozoa in the testis [26], 20 weeks in Korean native goats, and 24 weeks in Assam goats [22]. The first spermatozoa to appear in the tubular lumen or the ejaculated semen are indicators of puberty in males. The age of puberty in males varies in animals and even from breed to breed of the same species [8]. A number of spermatozoa in the tubular lumen, as seen in the histological findings of the current study, suggested that Black Bengal goats reached puberty at 6 months of age. In order to investigate spermatogenesis and determine the age at which the animal reaches puberty, it is imperative to know precisely when the first wave of spermatogenesis ends and the first spermatozoa arrive in the testis. In the current investigation, we discovered the first spermatozoa in the seminiferous tubule lumen of 6-month-old goats. The probability of spermatozoa appearing after 4 months and before or at 6 months of age could not be determined in the present work because the investigation was conducted at 4 and 6 months. To pinpoint the precise moment that the first wave of spermatogenesis ended and the first spermatozoa appeared in the tubules of the testis in Black Bengal goats, further thorough research is required.

## Conclusion

The structural development of the testis, or the age at which puberty begins in Black Bengal goats, varies from other documented goat breeds and is species-specific. Our results demonstrated that a notable increase in the diameter of seminiferous tubules, luteinization of seminiferous tubules, and stratification of the seminiferous epithelium in seminiferous tubules in Black Bengal goats occurred in the 4th month when the initiation of spermatogenesis occurred. The development of seminiferous tubules was complete, i.e., all spermatogenic cell types were revealed in the seminiferous tubules by the 6th month of postnatal age. By the age of 6 months, Black Bengal goats had established spermatogenesis, or the beginning of puberty, as shown by the development of a significant number of spermatozoa in the lumen of seminiferous tubules or stuck to the ad luminal border of the Sertoli cells. However, further investigation is needed to fully understand quantitative histology, such as stereology, in order to estimate the number of cells in the testis and pinpoint the exact moment that the first wave of spermatogenesis was completed and the first spermatozoa appeared in the testis of Black Bengal goats.

## List of Abbreviations

μm, micrometer; cm, centimeter; d, day; gm, grams; m, month; n, number; w, week.
